# Role of podocalyxin in astrocytoma: Clinicopathological and *in vitro* evidence

**DOI:** 10.3892/ol.2013.1556

**Published:** 2013-09-02

**Authors:** TIANXIANG HUANG, XIN JIN, LEI HE, MINGYU ZHANG, JUN WU, YANJIN WANG, JIASHENG FANG

**Affiliations:** 1 Department of Neurosurgery, Xiangya Hospital, Central South University, Changsha, Hunan 410008, P.R. China; 2 Department of Emergency, The Third Xiangya Hospital, Central South University, Changsha, Hunan 410013, P.R. China

**Keywords:** astrocytoma, podocalyxin, overall survival, disease-free survival, Ki-67, temozolomide, cell survival, Akt

## Abstract

The present study examined the expression of podocalyxin (PODX) in surgically-resected astrocytomas, associated the levels of PODX expression with the clinicopathological characteristics and survival outcomes of astrocytoma and assessed how PODX affected the viability of astrocytoma cells following the administration of chemotherapeutic agents. The immunohistochemical analysis of 102 patient samples revealed that a high expression of PODX was significantly associated with high-grade astrocytomas (P<0.001) and a high Ki-67 labeling index (LI; P<0.001). A Kaplan-Meier survival analysis demonstrated that the high PODX expression group had significantly shorter disease-free survival (DFS) and overall survival (OS) rates compared with the low expression group (P<0.001). The multivariate analysis using the Cox’s proportional hazards model revealed that a high expression of PODX, a high World Health Organization grade and a high Ki-67 LI were independent factors for shorter DFS and OS times. A subsequent *in vitro* study using SW1783 and U-87 human astrocytoma cell lines revealed that knocking down PODX decreased astrocytoma cell viability against temozolomide-induced apoptotic stress through the inhibition of the Akt survival signaling pathway. In conclusion, the *in vivo* findings indicated that a high expression of PODX is predictive of a poor survival outcome and, thus, may be used as a prognostic factor to predict the survival outcomes of astrocytoma patients. The *in vitro* findings indicated that PODX may promote astrocytoma cell viability against chemotherapeutic agent-induced apoptotic stress through the Akt pathway, indicating that PODX may be a novel target for overcoming chemoresistance in astrocytomas.

## Introduction

Mainly composed of neoplastic astrocytes and accounting for 80–85% of all gliomas, astrocytomas are staged as low to high grades (grades I–IV, respectively) on the basis of nuclear atypia, endothelial hyperplasia, mitotic activity and necrosis (1). Each year in the United States, >51,000 individuals are diagnosed with primary tumors of the brain. For those with astrocytomas, nearly 75% succumb to the disease within five years of diagnosis (2). The mortality rate for astrocytomas remains high, although the use of surgery, radiation and chemotherapy have improved the length of survival. This underscores the requirement to understand the clinicopathological factors underlying the prognosis for patients with this disease (2).

Podocalyxin (PODX) is a transmembrane protein that is expressed by a number of human cell types, including hematopoietic progenitors, platelets and vascular endothelial cells (3). Increased PODX expression has been associated with a subset of aggressive cancers, including acute myeloid and lymphoid leukemia, myeloid sarcomas and certain breast, liver, pancreatic and kidney tumors (3,4). The clinical significance of PODX in cancer progression has been investigated in numerous types of tumors, including breast, colon and uterine carcinomas. In uterine endometrioid adenocarcinomas, PODX expression is correlated with the tumor grade (5), while the overexpression of this protein is an independent indicator of a poor outcome in breast and colorectal carcinomas (6,7). PODX also reportedly leads to an increased activation of phosphatidylinositol 3-kinase (PI3K) activity in breast cancer and prostate cancer cells (8). Therefore, PODX is a candidate for playing a critical role in cancer development and aggressiveness. A previous study detected PODX expression in 42.9% of the anaplastic astrocytoma samples tested and 54.8% of the glioblastoma samples, indicating that PODX may be associated with the malignant progression of astrocytic tumors (9). However, to date, the role of PODX in astrocytoma progression remains unclear. The present study examined the expression of PODX in surgically-resected astrocytomas, associated the level of PODX expression with the clinicopathological characteristics and survival outcomes of astrocytomas and assessed how PODX affected the viability of astrocytoma cells treated with chemotherapy agents.

## Materials and methods

### Patients

Surgically-resected human astrocytoma samples from 102 patients who were treated at the Department of Neurosurgery of Xiangya Hospital, Central South University (Changsha, Hunan, China) were collected. These tumors were excised from 55 males and 47 females, with an age range of 6–82 years and a mean age of 44 years at diagnosis. The tumors were classified according to the World Health Organization (WHO) criteria (10). The samples included 41 diffuse astrocytomas (WHO grade II), 30 anaplastic astrocytomas (WHO grade III) and 31 glioblastomas (WHO grade IV). None of the patients were administered radiotherapy, chemotherapy or immunotherapy prior to the surgery. All the patients underwent surgical intervention with a maximum safe resection of the tumors. In 89% of the cases (91/102), the surgery was described as a complete or almost complete macroscopic resection of the tumor. In 11 cases (four anaplastic astrocytomas and seven glioblastomas), a subtotal removal of the tumor was performed due to tumor invasion to eloquent areas. In the anaplastic astrocytoma and glioblastoma cases, post-operative radiotherapy and adjuvant chemotherapy were routinely added as follows: Post-operative radiotherapy (1.8–2.0 Gy/fraction, 60 Gy in total) plus concurrent daily chemotherapy (75 mg/m^2^/day temozolomide) for 42 days, followed by six cycles of temozolomide (200 mg/m^2^/day for five days, every 28 days). In the diffuse astrocytoma cases, only radiotherapy (40 Gy in standard) was added. Tumor recurrence was observed in 47 cases (one case of diffuse astrocytoma, 15 of anaplastic astrocytoma and all the glioblastoma cases). The evidence of recurrence was based on the radiological findings. This study was performed according to the principles set out in the Declaration of Helsinki 1964 and all subsequent revisions. Informed consent was obtained from all patients. Approval for this study was obtained from the Ethics Committee of Xiangya Hospital.

### Cells lines and reagents

SW1783 and U-87 human astrocytoma cell lines were purchased from the American Tissue Culture Collection (ATCC; Rockville, MD, USA). The human PODX short hairpin RNA (shRNA) plasmid (RHS3979-98487921) and the pLKO.1 empty plasmid (RHS4080) were purchased from Open Biosystems Inc. (Huntsville, AL, USA). Anti-PODX (3D3; 39-3800) antibody was purchased from Life Technologies (Carlsbad, CA, USA). Anti-Akt (ser473; sc-24500) and anti-P-Akt (ser473; sc-101629) antibodies were purchased from Santa Cruz Biotechnology, Inc. (Santa Cruz, CA, USA). All the secondary antibodies were purchased from Jackson ImmunoResearch Laboratories (West Grove, PA, USA). The 3-(4,5-dimethylthiazol-2-yl)-2,5-diphenyltetrazolium bromide (MTT) cell proliferation and viability assay kit was purchased from R&D Systems (Minneapolis, MN, USA). Temozolomide and all the reagent grade chemicals were purchased from Sigma.

### Immunohistochemistry

Formalin-fixed, paraffin-embedded tissues were cut into consecutive 4-μm sections. Hematoxylin and eosin staining was performed for the histological diagnosis. The immunostaining for PODX was performed using the streptavidin-biotin-peroxidase method. Briefly, the histological slides were deparaffinized in xylol. The slides were heated in 0.01 M citrate buffer for 10 min in a microwave oven. Subsequent to being cooled for 20 min and washed in phosphate-buffered saline (PBS), endogenous peroxidase was blocked with methanol containing 0.3% hydrogen peroxide (H_2_O_2_) for 30 min, followed by incubation with PBS for 30 min. The slides were then incubated overnight at 4°C with polyclonal rabbit anti-PODX (1:500) and stained using the avidin-biotin complex method (Vectastain^®^ ABC kit; Vector Laboratories, Inc., Burlingame, CA, USA). Coloration was developed using diaminobenzidine (DAB; Dako Diagnostics, Carpinteria, CA, USA) containing H_2_O_2_, and the sections were counter-stained with hematoxylin. In the negative control, the primary antibody was replaced by PBS. Two pathologists who were blinded to the clinical and pathological data independently examined the slides. A total of ten high-power (×400) view fields were selected from each sample and the PODX expression in tumor cells was scored based on the extent (relative number of PODX-positive cells) and intensity of the staining: +, 10–25% weakly to moderately stained cells; ++, 10–25% strongly stained cells; +++, 25–50% positive cells with moderate to strong staining; and ++++, >50% positive cells. Cohen’s κ coefficient was calculated to show the interobserver variability. The Cohen’s κ coefficient was 0.91 in this study. For the statistical analysis, the four grades of staining were reduced to two groups, low expression (+/++) and high expression (+++/++++). For the Ki-67 immunostaining, the MIB1 antibody (sc-101861; Santa Cruz Biotechnology, Inc.) was used as the primary antibody. The percentage of positively-stained cells among the total tumor cells that were counted in ten randomly picked high-power (×400) view fields was used as the Ki-67 labeling index (LI), an indicator of the tumor cell proliferative potential (11).

### Quantitative (q)PCR

RNA was prepared from the brain tissue samples using TRIzol reagent followed by purification using the TURBO DNA-free system (Ambion, Austin, TX, USA). The cDNAs were synthesized using SuperScript II Reverse Transcriptase (Invitrogen, Carlsbad, CA, USA). Quantitative PCR was performed on the LightCycler Thermal Cycler system using the SYBR Green I kit (Roche Diagnostics, Indianapolis, IN, USA) as described by the manufacturer. The results were normalized against that of the housekeeping gene, glyceraldehyde-3-phosphate dehydrogenase (GAPDH) in the same sample. The primers that were used were as follows: Human PODX forward, 5′-AATTCCTTTCCCAGTTGT-3′ and reverse, 5′-TTCTCAGTAAATTCCAGTGTA-3′; and human *GAPDH* forward, 5′-GACTCATGACCACAGTCCATGC-3′ and reverse 5′-AGAGGCAGGGATGATGTTCTG-3′. Each experiment was repeated twice in triplicate.

### Lentiviral transduction

The PODX shRNA lentiviral particles contained expression constructs encoding target-specific shRNA that were designed to specifically knockdown PODX gene expression. The control shRNA lentiviral particles contained a scrambled shRNA sequence that would not lead to the degradation of any cellular mRNA and was used as a negative control for the PODX shRNA lentiviral particles. Lentiviral transduction was performed in the SW1783 and U-87 cells. Pools of stable transductants were generated via selection with 5 μg/ml puromycin.

### In vitro cell viability assay

*In vitro* cell viability was determined using the MTT cell proliferation and viability assay kit as described by the manufacturer (R&D Systems). Briefly, the cells were cultured at 15×10^3^ cells/well in 96-well tissue culture plates and incubated at 37°C for 8 h with or without 100 μM temozolomide. At the end of the culture period, the cells were washed with PBS, the MTT reagents were added according to the manufacturer’s recommendations and the absorbance was measured at 570 nm using an enzyme-linked immunosorbent assay (ELISA) plate reader. The proliferation/viability of the cells that were stably transduced with scramble control shRNA or PODX-shRNA was expressed as a fold change to that of the normal control cells (designated as 1). Each experiment was repeated three times in triplicate.

### Western blot analysis

Immunoblotting was performed using the respective antibodies. Briefly, the cells were dissolved in 250 μl 2× sodium dodecyl sulfate (SDS) loading buffer [62.5 mm Tris-HCl (pH 6.8), 2% SDS, 25% glycerol, 0.01% bromophenol blue, 5% 2-mercaptoethanol] and incubated at 95°C for 10 min. Equal amounts of the proteins for each sample were separated by 10% SDS-polyacrylamide gel and blotted onto a polyvinylidene difluoride microporous membrane (Millipore, Billerica, MA, USA). The membranes were incubated for 1 h with a 1/1,000 dilution of primary antibody and washed and revealed using secondary antibodies with horseradish peroxidase conjugate (1/5,000; 1 h). Peroxidase was revealed using a GE Healthcare ECL kit (Little Chalfont, Buckinghamshire, UK). The proteins were quantified prior to being loaded onto the gel, and the equal loading of the extracts was verified by Ponceau coloration.

### Statistical analysis

The statistical analyses were performed using SPSS for Windows 10.0 (SPSS, Inc., Chicago, IL, USA). The data values were expressed as the mean ± SD. The associations between PODX expression and the various clinicopathological variables were analyzed using a χ^2^ test. The comparison of Ki-67 LI between the PODX low and high expression groups was performed using Student’s t-tests. The two end-points examined for the survival analyses were disease-free survival (DFS) and overall survival (OS). OS was defined from the day of surgery until the day the patient succumbed. The data from the patients who had survived until the end of the observation period were censored at their last follow-up visit. Succumbing to a cause other than astrocytoma or survival until the end of the observation period was considered a censoring event. DFS was defined from the end of primary therapy until the first evidence of local, regional or distant tumor progression of the disease, if the patient revealed no evidence of disease following primary therapy. DFS and OS curves were plotted for the PODX low and high expression groups using the Kaplan-Meier method. A log-rank test was employed to compare the survival curves. The Cox proportional hazards model was used for the multivariate analysis. The statistical significance level of this study was set at a two-tailed α=0.05.

## Results

### Association of PODX expression with clinicopathological variables in astrocytomas

**Figure 1:**
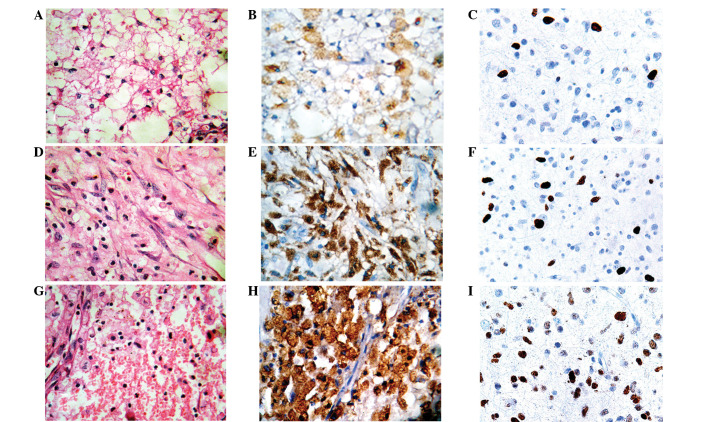
Immunohistochemical staining for podocalyxin (PODX) and Ki-67 in astrocytomas. Immunostaining for PODX (brown color) was observed in World Health Organization (WHO) grade (B) II, (E) III and (H) IV astrocytomas. Immunostaining for Ki-67 (brown color) was observed in WHO grade (C) II, (F) III and (I) IV astrocytomas. All the slides were counterstained with hematoxylin. The histotological images for WHO grades (A) II, (D) III and (G) IV astrocytomas are also shown. All images are ×400 magnification.

Immunohistochemical staining for PODX and Ki-67 was performed in samples of WHO grade II, III and IV astrocytomas (Fig. 1). PODX protein was mainly expressed in the cytoplasm and cell membranes of the astrocytoma cells, while Ki-67 staining was observed in the nuclei (Fig. 1). Only low PODX expression was detected in the tumor cells in 12.2% (5/41) of the grade II astrocytoma samples. High PODX expression was detected in the tumor cells in 90.3% (28/31) of the WHO grade IV tumors, 50.0% (15/30) of the WHO grade III tumors and none (0/41) of the WHO grade II tumors (Table I). qPCR analyses revealed that high-grade astrocytomas demonstrated higher PODX mRNA levels than low-grade astrocytomas (Table II). As shown in Table I, compared with the low expression group, a high expression of PODX was significantly associated with the high-grade astrocytomas (P<0.001). Among the patients with a high expression of PODX, 97.7% (42/43) had a Ki-67 LI of ≥10, which occurred in only 17.4% (4/23) of the patients with a low expression of PODX (P<0.001). Consequently, the high PODX expression group demonstrated a significantly higher Ki-67 LI than the low expression group (16.95±5.87 vs. 6.62±5.20; P<0.001). Furthermore, while tumor recurrence was observed in 97.7% (42/43) of the patients with a high expression of PODX, only 21.7% (5/23) of the patients with a low expression of PODX demonstrated tumor recurrence (P<0.001).

### Survival analysis

**Figure 2:**
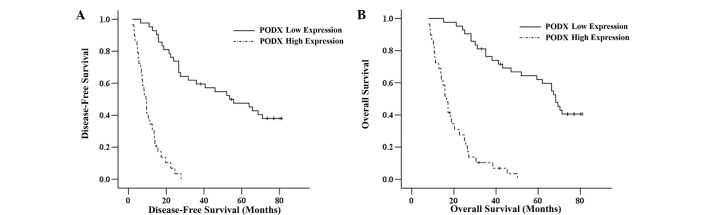
Kaplan-Meier curves for disease-free survival (DFS) and overall survival (OS) in astrocytoma patients. (A) DFS and (B) OS curves were plotted for podocalyxin (PODX) low and high expression groups using the Kaplan-Meier method. Log-rank tests were performed to compare the survival curves and revealed that a high expression of PODX was significantly associated with a short DFS and OS (P<0.001). Censored events are marked with vertical lines.

The Kaplan-Meier survival analysis revealed that the high PODX expression group had significantly shorter DFS and OS rates compared with the low expression group (P<0.001; Fig. 2). The multivariate analysis using Cox’s proportional hazards model revealed that a high expression of PODX, a high WHO grade and a high Ki-67 LI were independent factors for shorter DFS (Table III) and OS (Table IV) times.

### Effect of knocking down PODX on cell viability against temozolomide-induced apoptotic stress in astrocytoma cells

**Figure 3:**
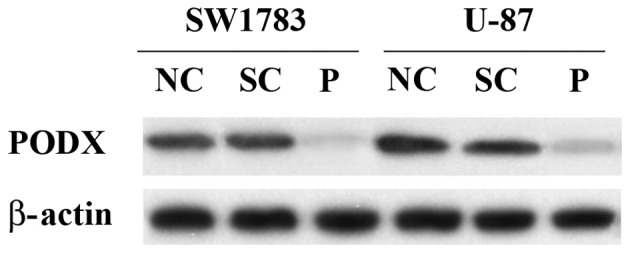
Knockdown of endogenous podocalyxin (PODX) expression in SW1783 and U-87 astrocytoma cells. The expression of PODX in normal control cells (NC), cells that were stably transduced with scramble control short hairpin RNA (shRNA; SC) and cells that were stably transduced with PODX-shRNA (P) was analyzed using western blot analysis. β-actin blotting was used as a loading control.

To explore the molecular mechanisms underlying the potential tumor-promoting effect of PODX on astrocytoma patients, shRNA was used to knockdown PODX expression in the SW1783 (grade III) and U-87 (grade IV) human astrocytoma cell lines. Western blot analysis demonstrated that the shRNA knocked down >75% of endogenous PODX expression in the SW1783 and U-87 cells (Fig. 3). By contrast, the scramble control shRNA exhibited no significant effect.

**Figure 4:**
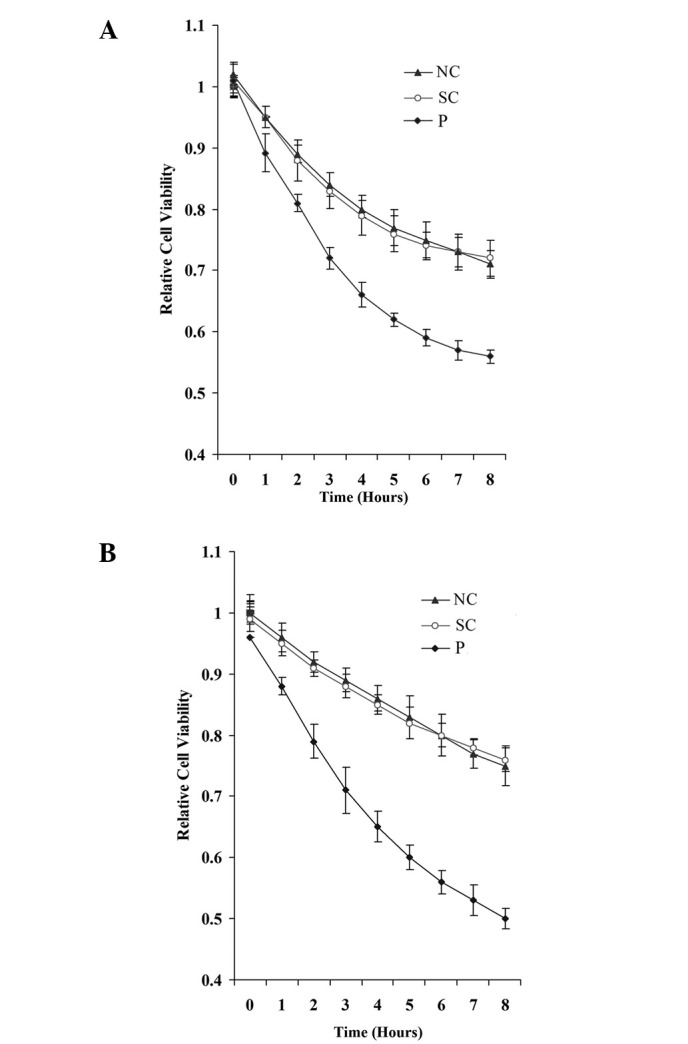
*In vitro* cell viability of SW1783 and U-87 astrocytoma cells that were treated with temozolomide. (A) SW1783 and (B) U-87 cells were treated with 100 μM temozolomide for 8 h. *In vitro* cell viability was determined using 3-(4,5-dimethylthiazol-2-yl)-2,5-diphenyltetrazolium bromide (MTT) assays each hour. The cell viability of the normal control cells (NC), the cells that were stably transduced with scramble control short hairpin RNA (shRNA; SC) and the cells that were stably transduced with podocalyxin (PODX)-shRNA (P) was analyzed. Cell viability is presented as fold changes to that of the normal control cells (NC; designated as 1).

The podocalyxin-like (PODXL) gene has been reported to promote the metastatic potential of tumor cells. As tumor cell survival is critical for metastasis (12), the effect of PODXL was examined on astrocytoma cell viability against apoptotic stress. Knocking down PODX did not significantly alter the cell proliferation/viability in the normal culture conditions (data not shown). However, when the cells were treated with 100 μM temozolomide, an apoptosis-inducing chemotherapeutic agent that is used to treat high-grade astrocytoma, the knockdown of PODX significantly decreased cell viability in the SW1783 and U-87 cells (Fig. 4).

### Effect of knocking down PODX on the Akt survival signaling pathway in astrocytoma cells

**Figure 5:**
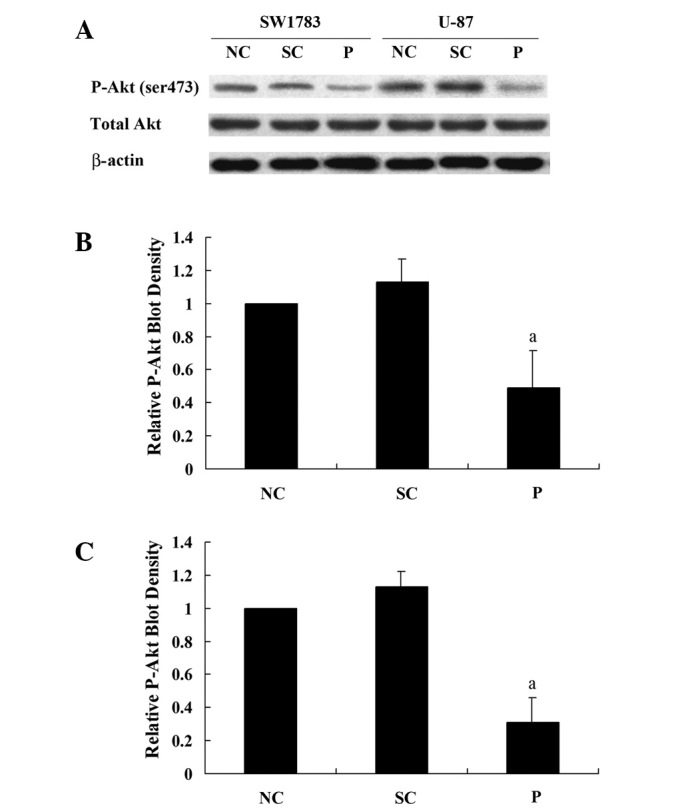
Western blot analysis of phosphorylated Akt (P-Akt) levels in podocalyxin (PODX)-knockdown astrocytoma cells. (A) In the SW1783 and U-87 cells, the levels of total Akt and P-Akt at serine 473 (ser473) in normal control cells (NC), cells that were stably transduced with scramble control short hairpin RNA (shRNA; SC) and cells that were stably transduced with PODX-shRNA (P) was determined using western blot analysis. β-actin blotting was used as a loading control. P-Akt (ser473), total Akt and β-actin blots were measured by densitometry. The density of the P-Akt (ser473) blot was normalized against that of total Akt and β-actin to obtain a relative P-Akt (ser473) blot density for (B) SW1783 and (C) U-87 cells, which was expressed as a fold change to the relative P-Akt (ser473) blot density of NC (designated as 1). ^a^P<0.05, vs. NC and SC.

As PODX demonstrated a protective effect on the astrocytoma cells against temozolomide-induced apoptotic stress, the effect of knocking down PODX on the Akt survival signaling pathway was analyzed. In the SW1783 and U-87 cells, knocking down PODX significantly decreased phosphorylation at serine 473 (ser473) of Akt, which is required for the full activation of Akt (Fig. 5). Taken together, these results indicate that PODX is able to increase the activation of the Akt signaling pathway and thereby astrocytoma cell viability against apoptotic stress.

## Discussion

PODX is implicated in a number of disease processes, including malignant progression (7,13). The present study explored the role of PODX expression in astrocytoma using patient samples and cell lines. A high expression of PODX was observed to be significantly associated with high WHO grade astrocytomas and was an independent factor for shorter DFS and OS times in the astrocytoma patients, indicating that PODX expression may serve as a predictive factor of a poor prognosis for astrocytoma patients. The *in vitro* data indicated that knocking down PODX markedly inhibited the activation of the Akt survival signaling pathway and decreased cell viability against apoptotic stress in the astrocytoma cell lines.

A previous study detected PODX expression in 42.9% of anaplastic (grade III) and 54.8% of glioblastoma (grade IV) astrocytomas. In diffuse astrocytoma (grade II) samples, PODX expression was observed only in the vascular endothelial cells (9). In the present study, however, weak PODX expression was detected in the diffuse astrocytoma cells in a few samples (Table I). This may be due to the sample size of the diffuse astrocytomas, which was larger in the present study than in the previous study (n=41 vs. n=6, respectively), which led to an improved chance of detecting PODX expression in the diffuse astrocytoma cells. Previous studies have shown that PODX overexpression is a predictor of breast cancer progression (7) and that PODXL gene variants are associated with tumor aggressiveness (13). This is consistent with the present *in vivo* findings showing that a high expression of PODX was significantly associated with a high proliferative potential, as indicated by the Ki-67 staining in the astrocytoma tissues. Since the multivariate Cox’s proportional hazards model demonstrated that a high expression of PODX and a high Ki-67 LI were independent factors for shorter DFS and OS times in astrocytoma patients, proliferation is unlikely to be the sole mechanism of PODX signaling.

PODX is a candidate for playing a critical role in cancer aggressiveness and malignancy (9). As cancer cell survival is critical for cancer aggressiveness (12), grade III (SW1783) and grade IV (U-87) astrocytoma cell lines were used to explore the protective effect of PODX on high-grade astrocytoma cells against temozolomide-induced apoptotic stress. Endogenous PODX was knocked down rather than overexpressed in the astrocytoma cells, since overexpression is prone to generating artifacts. Temozolomide alkylates/methylates DNA, which damages the DNA and triggers the death of tumor cells (14). Borges *et al*(15) revealed that the IC_50_ of temozolomide on glioblastoma cells was >300 μM. Thus, in the present study, a relatively small concentration of temozolomide (100 μM) was used to induce apoptotic stress without killing the majority of the cells. The present results demonstrated that PODX was able to promote astrocytoma cell viability against apoptotic stress induced by temozolomide through the Akt survival signaling pathway. The findings not only provide *in vitro* evidence for the tumor-promoting role of PODX in astrocytomas, but also indicate that PODX is significant for the development of chemoresistance in astrocytomas.

In conclusion, the *in vivo* results indicated that a high expression of PODX is predictive of a poor survival outcome and therefore, may be used as a prognostic factor to predict the survival outcomes of astrocytoma patients. The *in vitro* findings indicate that PODX is able to promote astrocytoma cell viability against chemotherapeutic agent-induced apoptotic stress, indicating that PODX may be a novel target for overcoming chemoresistance in astrocytomas.

## Figures and Tables

**Table I:** Association of PODX expression with clinicopathological variables in astrocytomas.

	PODX expression in tumor cells		
	
Variable	Low	High	P-value
Total, n	23	43	
Gender, n			
Male	10	27	0.13
Female	13	16	
Age, n			
≥50 years	9	12	0.35
<50 years	14	31	
Mean Ki-67 (LI±SD)	6.62±5.20	16.95±5.87	<0.001^a^
≥10%, n	4	42	
<10%, n	19	1	<0.001^a^
WHO grade, n			
II	5	0	
III	15	15	
IV	3	28	<0.001^a^
Tumor recurrence, n			
+	5	42	
−	18	1	<0.001^a^

a P<0.05.

PODX, podocalyxin; LI, labeling index; WHO, World Health Organization.

**Table II:** qPCR analysis of relative mRNA levels of PODX in various grades of astrocytoma.

WHO grade	n	Relative PODX mRNA level
II	41	77.80±80.39
III	30	256.±140.05^a^
IV	31	857.26±549.43^a^,^b^

Association between PODX expression and various clinicopathological variables were analyzed using the χ^2^ test. Comparison of Ki-67 LI between PODX low and high expression groups was performed using Student’s t-test.

a P<0.05 vs. grade II;

b P<0.05 vs. grade III.

PODX, podocalyxin; LI, labeling index; WHO, World Health Organization; qPCR, quantitative PCR.

**Table III:** Cox’s proportional hazards analysis for DFS of astrocytoma patients.

Variable	Relative risk (95% CI)	P-value
Gender		
Female	1.0 (reference)	
Male	1.35 (0.85–2.28)	0.31
Age, years		
<50	1.0 (reference)	
≥50	1.72 (0.73–3.65)	0.19
Ki-67 LI, %		
<10	1.0 (reference)	
≥10	9.61 (3.71–23.70)	0.011^a^
WHO grade		
IV vs. II	201.53 (46.95–1012.87)	<0.001^a^
IV vs. III	9.22 (2.97–24.85)	<0.01^a^
PODX expression		
Low	1.0 (reference)	
High	9.74 (5.19–20.16)	<0.01^a^

a P<0.05.

WHO, World Health Organization; CI, confidence interval; LI, labeling index; PODX podocalyxin; DFS, disease-free survival.

**Table IV:** Cox’s proportional hazards analysis for OS of astrocytoma patients.

Variable	Relative risk (95% CI)	P-value
Gender		
Female	1.0 (reference)	
Male	1.62 (0.95–3.69)	0.23
Age, years		
<50	1.0 (reference)	
≥50	1.90 (0.89–5.75)	0.14
Ki-67 LI, %		
<10	1.0 (reference)	
≥10	7.75 (2.63–18.37)	0.017^a^
WHO grade		
IV vs. II	179.05 (27.42–982.56)	<0.001^a^
IV vs. III	6.93 (3.03–19.31)	0.031^a^
PODX expression		
Low	1.0 (reference)	
High	6.96 (3.25–15.48)	0.035^a^

a P<0.05.

LI, labeling index; WHO, World Health Organization; PODX, podocalyxin; CI, confidence interval; OS, overall survival.
